# Network learning and transitional change in a global project for transforming sustainability education

**DOI:** 10.12688/openreseurope.14407.1

**Published:** 2022-02-18

**Authors:** Martin Melin, Geir Lieblein, Tor Arvid Breland, Charles Francis

**Affiliations:** 1 Department of People and Society, Swedish University of Agricultural Sciences, Box 190, 234 22 Lomma, Sweden; 2 Department of Plant Science, Faculty of Biosciences, Norwegian University of Life Sciences, P.O Box 5003, 1430, Ås, Norway; 3 Department of Agronomy and Horticulture, Institute of Agriculture and Natural Resources, 279 Plant Science Building, University of Nebraska –Lincoln, UNL, Lincoln, Nebraska, 68583-0910, USA

**Keywords:** Action learning, education, systems thinking, educational transformation, agrifood system

## Abstract

Educational strategies globally are changing from an authoritative, top-down model to one focused on greater student and stakeholder participation and collaborative involvement in planning and implementation of educational activities. In addition to emphasis on student-centered education, strategies are evolving to encompass learning organizations and learning networks. These are essential to address the complexity and scope of tomorrow’s challenges, involving issues that could be called ’wicked problems’ not easily addressed by single disciplines nor resulting in solutions that please all the players. Such incommensurate problems make it essential to tap into all possible sources of information, to explore multiple learning strategies for solutions, and to seek answers that will be acceptable to all those impacted. Such an approach includes knowledge co-production, and methods of co-learning to reach mutually acceptable outcomes. Meaningful transitions or transformations require attention to creative network organisation, learning through practical action, cooperation and mutual respect among participants, comfortable and meaningful activities with stakeholders, agreed-upon structures and practices, agreed-upon outcomes and methods to reach them, as well as shared commitment to complete tasks and share of credit for accomplishing them. The NEXTFOOD Network is used as an example of how these goals were set, and how a transformation through their implementation is playing out. A successful transition involves ownership by all the players, meaningful and lasting changes in roles of various players and their institutions, willingness to experiment with new methods, and shared responsibility for outputs and impacts. NEXTFOOD Network partners are making concerted efforts to overcome institutional, disciplinary, and long-established barriers to this type of transformation in education, and have dealt with the unique pandemic challenges to creating a meaningful transition that will emerge as a resilient and pro-active strategy for the continual improvement of cooperative education.

## Plain language summary

The sustainability challenges of agriculture and food production, such as accelerating climate change and the immense loss of biodiversity, are of the kind that they cannot be adequately addressed by single disciplines only and cannot be solved to the satisfaction of all the players. Dealing with such problems will require learning approaches that builds on many sources of knowledge, as well as adopting proven practices learned from farmers and other practitioners with long experience.

NextFood is a tweleve-country collaborative network that aims at a fundamental change towards more sustainable food production, by bringing together university students, academics, field professionals, farmers and other stakeholders in order to create a community of learning from experience and research. In addition to introducing new teaching methods into their own courses and programs, innovative teachers meet regularly to share experiences so that the collaborative network itself becomes a learning community. The question this research study set out to answer is how learning and collaboration in such a global collaborative network can be facilitated in order to contribute to a fundamental change of the way education is done at universities today.

Based on experiences of the network members, we have learned about successes as well as difficulties in implementing new approaches for sustainability education at universities. Lessons learned have been shared in the consortium workshops, and can be used by other project networks in education as well as other fields for planning and adjusting strategies for change: meaningful transitions or transformations require attention to creative network organisation, to learning through practical action, to cooperation and mutual respect among participants, to comfortable and acceptable activities with stakeholders, to agreed-upon structures and practices, to agreed-upon outcomes and methods to reach them, and to shared commitment to complete tasks and share the credit for accomplishing them.

## Introduction

The global scope of today’s sustainability challenges has raised awareness of the importance of different forms of collaborative networks, where diverse actors come together in various forms of partnerships to promote systems change. Despite coming from widely heterogenous backgrounds, cultures, and institutions these actors choose to engage in collaboration because they believe this strategy will help achieve goals that would be unattainable working on their own ( Camarinha-Matos, 2005; Dzhengiz, 2020). Global project networks bring together actors from academia, business, public, and non-profit sectors to form international consortia that span disciplines, sectors and nations to potentially accelerate research and learning on solutions to sustainability challenges ( Keeler *et al.,* 2016). In collaborative EU-projects, partners from diverse organisations and countries cooperate to address societal challenges through research. A recent study ( Vinke-de Kruijf *et al.,* 2020) on collaborative EU-projects concluded that wider learning outcomes cannot simply be achieved by internal project conditions, instead there needs to be increased focus on the processes of knowledge co-production and each projects’ interactions within the transition context. However, these interactive processes are not deeply understood, and how to organise and catalyse effective learning across various project domains deserves more focus. The need to foster collaborative approaches to improve governance of social-ecological systems was highlighted by Berkes (2017, p. 9) who concluded that “operationalizing adaptive governance requires learning from, and improving on, practices of collaborative learning”.

Literature on strategic niche management has emphasized the importance of learning from local experiments with novel ideas ( Hoogma *et al.,* 2017; Kemp *et al.,* 1998). Project managers can study how learning outcomes from niche-experiments can complement each other and contribute to enhancing a global sustainability trajectory, based on a flow of knowledge and experience among individual experiments, and one emergent property may be general lessons drawn from the collective experience ( Geels & Deuten, 2006). In agricultural and forestry education, pioneering teaching practitioners leading local transdisciplinary education initiatives may contribute substantially to sustainability transition by engaging in processes of co-learning with other groups of teachers practicing similar educational strategies. By connecting with networks of educational pilot projects through action learning they may create opportunities for right-scaling and promoting alternative methods of sustainability education.

### Network learning in sustainability transitions

Transitions are large-scale radical changes in societal systems, often over a long period of time. They are triggered by innovation and external changes in society that put the dominant structure and institutions under pressure. Transitions are often related to solving complex sustainability challenges involving co-evolution of several different domains including technology, the economy, and education ( Loorbach & Rotmans, 2010; Rotmans *et al.,* 2001). Changes in education to assure that future professionals in the agricultural sector have the right set of sustainability skills and competences need to co-evolve with the agri-food systems and impact the way food is being produced and consumed. An understanding of such dynamic processes opens the possibility to manage sustainability transitions, or at least give them a nudge in the right direction. Several established learning traditions may be relevant for transition studies ( Van Mierlo & Beers, 2020), but network learning has not previously been mentioned in this context as far as we can determine.

Interorganizational networks represent a fourth system-level learning entity at a larger scale than individual, group and organization ( Knight, 2002). Network learning can be understood as “learning by a group of organisations as a group” ( Knight & Pye, 2005, p. 371). The concept of network learning is best applied to learning within an interorganizational setting, whether by individuals, groups, or organizations. In a network-centered view, changed network-level properties, such as network-level interpretations, practices, and structures, are the outcomes of the learning transition process ( Knight & Pye, 2005). Socio-technical regimes, such as the educational system, consist of networks of actors, including producers, end-users, public authorities, researchers, and financial institutions who through their coordinated actions most often contribute to the continuation of the mainstream way of doing things ( Geels, 2002). Calling for a new mode of sustainability education means challenging assumptions and ideas of ‘good educational practices’ engrained by experience and tradition in students, faculty and members of university management. The study of learning in a regime or emerging niche is especially relevant for addressing the interactions between actors. Network learning is different from the kind of organizational learning that is the result of inter-organisational partnerships and alliances ( Dzhengiz, 2020), where focus is on learning that takes place in each organization and not in the shared space of network members. For example, if learning processes result in changes within an organization without having any discernible effect on network properties, then one could say there has been organizational learning within the network ( Crossan *et al.,* 1995). Expanding this concept, ‘organisational learning’ is valuable when it affects how organisations learn to change their practices due to frequent interactions among its members. But in order to understand learning in sustainability transitions it is important to examine the interactions that occur between organisations through processes of learning, feedback and knowledge generation ( Van Mierlo & Beers, 2020). Moreover, network learning is associated with developing shared meaning among actors from different backgrounds and practices. This is a feature shared with transition management where stakeholders are organized in learning arenas to resolve differences in meanings and to co-create transition agendas ( Loorbach & Rotmans, 2010).

### Background and structure of the NEXTFOOD project

As a response to the need for innovation in transition education, a 12-country initiative funded by EU through the Horizon 2020 program, our NEXTFOOD (NF) project ( https://www.nextfood-project.eu/) was initiated to bring together teaching practitioners in several countries to co-create a future roadmap for education in agriculture and foods. Network members are researchers and teaching practitioners from diverse disciplines such as agroecology, social sciences, food studies and farming systems, as well as representatives from non-governmental organizations (NGOs) and business networks. A learning arena was established on the network level, focusing on testing new strategies, evaluating their successes, and providing a foundation for impacting the wider educational sector through organizing a broad participatory co-learning process.

The aim of this study was to examine the extent to which network learning occurred and to identify what learning processes were involved when effective facilitation was applied to an international network of teaching practitioners working to co-create a future roadmap for transformative education in agriculture, foods and forestry. The aim was also to identify challenges to the transformation of a collaborative global network and to suggest practical solutions for how these challenges can be overcome.

The network of teaching practitioners represented organisations seeking an effective transition of agricultural and forestry education within in a 12-country collaboration—the NEXTFOOD project— funded by EU through the Horizon 2020 programme to co-create a future roadmap for innovative, transformative education in agriculture, foods, and forestry. Partnering organisations were the Swedish University of Agricultural Sciences, Lund University and Skogforsk, Sweden; University of Oradea, Romania; University of South Bohemia České Budějovice and Bioinstitut, Czech Republic; Norwegian University of Life Sciences, Norway; American Farm School, Agronutritional Consortium of the Region of Central Macedonia and International Hellenic University, Greece; University of Bologna, International Center for Advanced Mediterranean Agronomic Studies and University of Gastronomic Sciences of Pollenzo, Italy; Deutsche Welthungerhilfe, Germany; Sekem Development Foundation, Egypt; Mekelle University, Ethiopia; ISEKI-Food Association, Vienna; Roskilde University, Denmark; University of Chile, Chile. Network members are researchers and teaching practitioners from a diversity of disciplines such as agroecology, social sciences, food studies, farming systems, and forestry, as well as representatives from NGOs and business networks. On the network level, a learning arena was established with focus on testing new strategies, evaluating their successes, and providing a foundation for impacting the wider educational sector. On this arena, the facilitation of a broad, participatory, action-oriented co-learning process took place to ensure that research outcomes and practical experience gained within working groups of educational pilot projects were circulated between different project domains. One goal was to promote institutionalizing transformation within the partnering organisations. Beyond local change the project had the goal of ‘broadening’, which according to transition theory is a key mechanism by which experiments in multiple contexts collectively contribute to an emerging change in culture, practice, and structure ( Van der Bosch & Rotmans, 2008).

In this study, we draw on network learning theory in an analysis of the adaptation of innovative educational practices, as imbedded in multiple cultures and academic structures. These include distinctive network domains in the NEXTFOOD research and innovation project, where we hope to increase the understanding of how collaborative projects can be managed to enhance capacity building among partners and promote broadening of promising initiatives in education, and ultimately promote a model for transition towards sustainability in other sectors. Practical applications of this study include management strategies for facilitators and project leaders involved in collaborative projects aiming to achieve broad societal impact.

## Methods

### Ethics statement

The research has been conducted under the ethics requirements and guidelines of the NextFood project (Deliverables 8.1, 8.2 and 8.3), which complies with regulation (EU) no 1291/2013 of the European Parliament and of the Council. A data management plan was developed to ensure that data collection and processing was performed in accordance to the GDPR. Written informed consent for publication of qualitative research data was obtained from the consortium members prior to participation.

### Approach

The action research approach described here was informed by several workshops organized at annual consortium conferences during the first three years of the project (May 2018–May 2021). Action research is a cyclical process where knowledge generation is combined with an active engagement of researchers in solving complex societal problems ( Levin & Ravn, 2007). In addition, our analyses build on observations made at meetings organized for consortium members in between the annual conferences. The workshops were facilitated by researchers in the NEXTFOOD project. The first workshop was organized in Malmö, Sweden in May 2018 to generate a mutual understanding of baseline conditions. Qualitative data were collected including viewpoints of faculty members and teaching professionals about project aims, ambitions, and consortium organization as well as barriers and opportunities for implementing research tasks of the project. These included reaching accord on learning achievements for the conference. The same assessment was done at three following conferences in Budweis, Czech Republic in June 2019, and two conferences organized online in June 2020 and in May 2021. At the on-line conferences we used the Zoom ( https://zoom.us/) meeting platform. Using NVivo software ( NVivo Limited, 2018) we applied content analysis to the data collected from workshops. The empirical data consisted of group-level workshop notes, i.e., each note was the result of several consortium members thinking together. Transcripts were coded using 18 different codes that emerged while elaborating the coding process. Coding was thematically organized in challenges, learning outcomes, and possible solutions emerging from the anticipated educational transformation. The codes were: challenges teacher role, challenges students, challenges stakeholders, challenges organisation, challenges project management, challenges coordination, challenges communication, challenges common understanding, challenges action learning, learning outcomes interpretations, learning outcomes network, learning outcomes institutions, learning outcomes process, learning outcomes coordination, solutions institutional change, solutions coordination, solutions communication, solutions action learning. Codings for learning outcomes were further divided into the following predefined sub-themes: network practices, structures, and interpretations following methods of Knight & Pye (2005). In this co-learning environment, it is appropriate that the methodology and specific activities also evolve as the project moves ahead.

**Figure 1.**
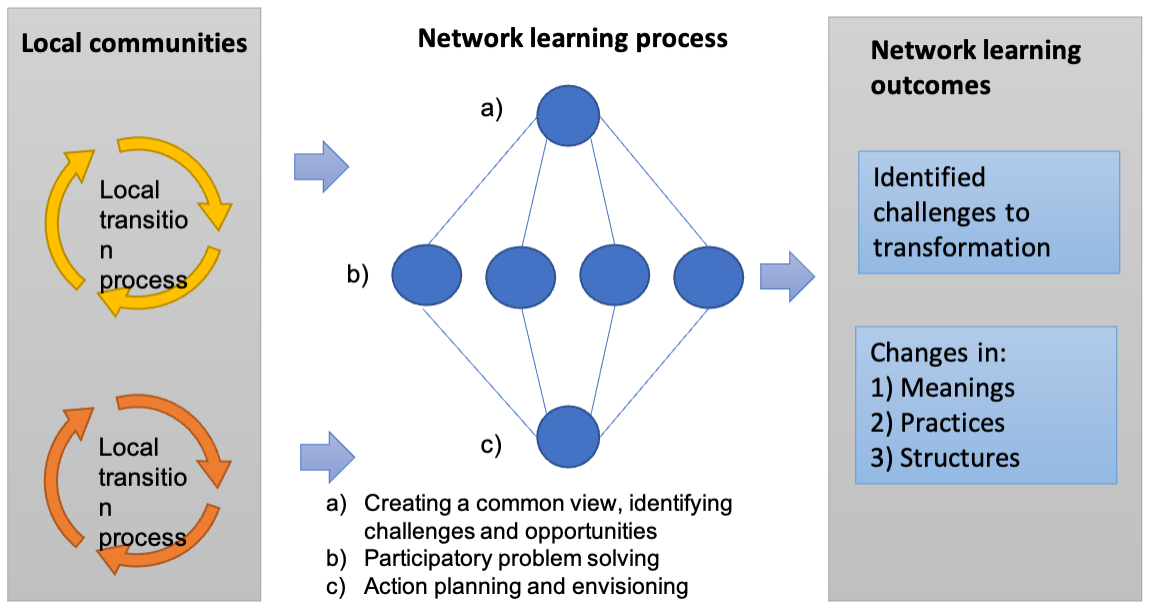
The consortium members engaged in a process governed by the NEXTFOOD action research model. In this model, a three-step consortium action learning cycle was linked to several multistakeholder research activities and local learning arenas run in parallel.

***Facilitation of network learning in the NEXTFOOD consortium.*** At annual conferences, 4 in total, members of the consortium had opportunities to engage in a process of problem structuring, action planning, and co-learning. Educators in each educational case form learning communities, and the consortium becomes a supra-community with all the players contributing their experiences and lessons learned ( Figure 1). In between the annual conferences there were two meetings for case leaders and several peer-to-peer learning activities on-line.

The workshop design at annual meetings was similar each year. It was planned as a learning cycle, starting with collective generation of an overall view of the project, then going into more detailed discussions about project matters on team and task level, and finally assembling the pieces into an action plan for the coming year. By engaging in dialogue, consortium members together developed agreed-upon meaning of aims and the overall project vision. We deliberately had few lengthy presentations at the meetings, which we thought would harm the opportunity for everybody to fully participate. The same transformation as we tried to achieve in the classrooms of the educational projects, i.e., a shift towards learning based on dialogue and participation, we also sought to achieve among ourselves in the consortium network. The following methods and tools were used at meetings:

Catch-up session

At each annual conference, 4 in total, a catch-up sessions was organized with the purpose to create a connection to the previous annual partner conference as well as to the ‘in-between’ activities. At the catch-up session all consortium members, about 50–60 people, had the opportunity to exchange ideas about the common goals of the project and to provide guidance for activities in the different work packages. The session was highly interactive, lasted for 1–2 hours and was facilitated by members of the project management.

Timeline

One method we found useful is to create a timeline for the project using a large roll of wallpaper, where all the project activities and outputs were noted, starting at the project kick-off until now. The paper was put the on the wall for the whole meeting and was used during the rest of the meeting whenever fruitful. This method was also used at one of the on-line meetings, but instead of sketching on brown wallpaper, workshop participants used a virtual whiteboard for co-creating a timeline.

‘Prouds and sorries’

The purpose of this exercise was to reflect on successes and disappointments related to the project work in the past year, and the participants were asked to note down their ‘prouds’ and ‘sorries’. The idea was to bring up challenges and work as a group to come up with ideas to solve them.

Guided dialogue

In a dialogue facilitated by members of the project management team, participants reflect on workshop questions first individually, then in small groups, and finally some highlights from each group are shared in a plenary session.

Work-in-progress

Teams provide a short presentation on work-in-progress, present what has been achieved during the past year, and some concerns about the process and the outcomes. This is then discussed by the larger group where colleagues are asked to come up with solutions and suggestion on how to tackle the challenges that are brought to the table.

Action planning

This is followed by a detailed planning of project activities by each team, resulting in a six-month action plan with a clear timelines and responsibilities.

Visioning

In one of the meetings (held in Vienna for case leaders, October 2019), a session on visionary thinking was organized. In this activity, the participants are given the task to envision the impact on the food system that will be achieved by the end of this project. The groups draw sketches of their shared visions and present them for the audience who have an opportunity to ask clarifying questions.

Wrap-up-session

In this session participants explore what is achieved during the meeting and used that as the starting point for looking towards the nearest future of the project. Participants are asked to discuss the following questions: a) What are we now more clear about?; b) What are we still unclear about that needs to be quickly resolved?; and c) What are the implications for what we should prioritize during the next six months?

## Results

The empirical data underlying the analysis was collected at four consortium conferences held annually between 2018 to 2021 ( Table 1) ( Melin *et al.,* 2021). At the annual consortium conferences the members were asked to individually reflect, and then discuss in groups and in plenary a) what is most challenging to the development and implementation of new action-oriented educational approaches in higher education, b) what has been achieved since last the meeting, c) what was achieved during the meeting, and d) implications for the future. The results of the analysis is shown in Table 2.

**Table 1.** Location, date and total number of participants of the four annual conferences.

Location	Date	Number of participants
Malmö, Sweden	6 May – 4 May, 2018	48
Budweis, Czech Republic	28 May – 30 May, 2019	47
On-line	3 June – 5 June, 2020	80
On-line *	4 May – 25 May, 2021	26

*The activities and meetings of this conference were spread out during three weeks. Number of participants based on participants at the catch-up session only.

**Table 2.** Examples of workshop notes presented for each of the 18 codes, including the frequency count of the coding scheme (total number of codings). ( Melin *et al.,* 2021).

Coding scheme	Quotes extracted from workshop notes including questions (Q) and answers (A).	Reference to dataset	Total number of codings
Challenges-action learning	Q: What would it take to shift the mindset from a linear to a cyclical one? A: Even if one is interested in the circular approach the others you are with {teaching peers} may not be interested or understand.	Guided dialogue, kick-off, Malmö, 2018	39
Challenges -common understanding	Q: What are the main challenges for you in this project? A: {We need} agreement and some basic more common understanding on sustainable agro-food systems and agroecology.	Guided dialogue, kick-off, Malmö, 2018	14
Challenges - communication	Q: What have been the main difficulties (individually, in your team and in the consortium)? A: On a consortium level, the covid situation has brought challenges in terms of communication.	Catch-up session, on-line June 2020	26
Challenges -coordination	Q: What have been the main difficulties (individually, in your team and in the consortium)? A: As we are getting closer to the end {of the project}, it is necessary to integrate the components from different work packages.	Catch-up session, on-line June 2020	34
Challenges -external stakeholders	Q: How does Nextfood meet the multi-stakeholder requirement today? A: We already do a lot of things to involve stakeholders {…..} but that this involvement is sometimes quite passive or sort of one-way communication.	Catch-up session, on-line June 2020	17
Challenges - management	Q: What are most challenging with the NextFood model? A: To convince people who decide about economics and time allocation that there are resources enough for the re-organizing that is necessary to use this model.	Guided dialogue, kick-off, Malmö, 2018.	7
Challenges -organisation	Q: What are the main challenges for you in this project? A: How to find our individual place and role in the project?	Guided dialogue, kick-off, Malmö, 2018.	20
Challenges -students	Q: What are your sorries from the previous year? A: How to get a proper discussion going {among students}?	Catch-up session, Budweis, 2019	9
Challenges-teacher role	Q: What do you find most challenging cout the NextFood model? A: {The challenge is} to go from being a teacher to a facilitator.	Guided dialogue, kick-off Malmö, 2018	24
Network learning outcomes -coordination	Q: What are we now more clear about? A: Now we have a better overview of the whole project + synergies/ linkages between workpackages.	Wrap-up session, Budweis, 2019	10
Network learning outcomes -institutional change	Q: What are you most proud of from the previous year? A: The institutional support. The project gave me the occassion to implement action learning "officially".	Wrap-up session, Budweis, 2019	14
Network learning outcomes - interpretations	Q: What will it take for Nextfood to improve impact even more in education? A: Thera was a bit of discussion of how we define technical and soft skills {…..}, we all arrived to the conclusion that {…..} in this project it is very important to work on soft skills like collaboration or facilitation and so on.	Catch-up session, on-line, 2020	23
Network learning outcomes -network	Q: What are the main achievements? A: Its good to be able to expand our professional network with people who are concerned with similar things.	Catch-up session, on-line, 2021	20
Network learning outcomes -process	Q: What are the main achievements? A: From a consortium perspective, I think the peer learning groups related to the Nextfood case development and research process have been a good addition.	Catch-up session, on-line, 2021	15
Solution -action learning	Q: What are the implications for the future? A: Implement skills {action learning} with those who are ready.	Catch-up session, Budweis, 2019	13
Solutions - communication	Q: How can the collaboration of the NF consortium become even better? A: Peer learning meetings have been very good to share experiences, to have inputs, comments.	Wrap-up session, on-line, 2021	31
Solutions - coordination	Q: How can the collaboration of the NF consortium become even better? A: Workshops in teams with the cases, more direct contact in smaller rooms, in order to have a more direct cooperation.	Wrap-up session, on-line, 2021	20
Solutions – institutional change	Q: What would it take to institutionalize the NextFood model? A: using methods that provide more long-lasting networks or dialogues with other teaching institutions. Such as roundtables and workshops.	Catch-up session, on-line, 2020	8
Total			344

Action learning

When it comes to action learning, consortium members experienced several challenges in the initial phase of the project, such as resistance among students and peer-teachers and they felt insecure in applying an action-oriented approach in their teaching. Numerous barriers would need to be overcome by faculty: institutional pressure to stick to conventional teaching methods, difficulties in explaining the new learning model to teaching peers, and resistance among university administration and need to provide extra time for planning and running such a course. There was concern that all students may not be ready for this transformation and it is necessary to understand their attitudes toward their own learning. At the second partner conference in Budweis one year into the project, participants focused much less on challenges related to the implementation of action learning, indicating they had developed the necessary knowledge and found successful strategies to deal with these challenges. The COVID-19 pandemic which hit in 2020 raised major challenges to teachers using action-oriented learning approaches, but also offered opportunities to develop creative methods for on-line learning. At the consortium conferences in 2020 and 2021, case leaders reported positive experiences of having the courses on-line, but at the same time they worried that lack of interaction with peers and stakeholders in real life might decrease learners’ experiential abilities. In some partner countries the infrastructure for was not enough developed in the countryside to successfully run courses on-line.

Teachers’ role

A concern raised by consortium members was how faculty can be supported to grow from their role as a conventional teacher to becoming a learning facilitator. One issue that surfaced was the negative attitude toward action-oriented learning practices among teachers who, according to several members of the consortium, are not always interested in alternative learning methods or are not willing to go outside their comfort zones. One year into the project most of the partners were convinced about the benefits of the new learning models. There was some introspection when they considered that “academic teachers have to accept that they are not the only knowledge holders but they too can learn from other stakeholders” (Consortium conference in Malmö, Sweden, 2018, session: a shift of mindset). Our observations and conversations with consortium participants and other educators in our universities is that this transition is well under way, and more people every year are ‘buying into the concept’ of co-learning and accepting the reality of depending on a wide range of information resources beyond the personal expertise of teachers.

Collaboration

The complexity of the project made it difficult for network members to grasp the overall picture and to see how different tasks linked together, which caused some frustration among partners at the kick-off meeting in 2018. This discussion continued at the second partner conference in Budweis in 2019 where the challenge of coordinating activities between different work packages was brought up as a major challenge for collaboration. We realized there were differences among members in knowledge level and understanding of the project structure. Some members had a good overview while others asked for a clearer picture of the contents of each work package and wanted to develop a better understanding of the linkages between them. Assuring that all consortium members have insight into various parts of the project as well as the whole is necessary to foster good collaboration to create complementarity, and to avoid overlapping activities. The challenge of coordinating activities between various tasks and work packages continued throughout the project and even increased towards the end when different elements had to be integrated in the final outcomes. At the first online meeting in 2020 the conversation changed to difficulties of remote collaboration. One general impression among consortium members was that project meetings can to a great extent be on-line, saving time and reducing carbon footprint due to less travelling, but not entirely replace in-person interactions because informal meetings face-to-face are very important. Concrete, straight-forward issues could be dealt with online, but the online format proved to be less efficient when it came to communication about more complex and less well-defined issues.

Communication

One year into the project consortium activities, research tasks and local learning arenas were up and running and there was need to share experience and knowledge from different initiatives.

Much of the discussions at the second partner meeting in Budweis were focused on how to improve internal project communication. In general, partners wanted to improve the horizontal information exchange between people on different project tasks and between members engaged in the same task, and they identified the need to organize more virtual meetings between physical conferences. A project website ( https://www.nextfood-project.eu), newsletters, and social media (Facebook, Twitter, Youtube and Instagram) were used for external communication, but the online platform for internal use had very few visitors and little material was being uploaded. We learned that the initial resistance for using the platform was in part explained by lack of instructions; partners asked for a tutorial in how they could use this strategy for sharing information. Instead, Zoom and Microsoft Teams were experienced as good solutions for bi- and multilateral meetings. In 2020 all meetings were online, with which all consortium members agreed due to the COVID-19 pandemic dangers and restrictions on travel. 

Organisation

The complexity of the NF project was discussed at the kick-off meeting in May 2018, and in the notes from the workshop outcomes several consortium members expressed frustration with not being able to see a clear structure of the whole project, some describing it as “amorphous” ( Melin *et al.*, 2021). The consortium consisted of a wide and diverse network of actors in a system with interaction and exchange between both actors within the system (among consortium members) and in the external educational landscape. Due to the complexity of the project, consortium members found it challenging to understand project structure and plans, including responsibilities, coordination of activities, and the process for moving forward. These worries were connected to the start of the project and at the consortium conference in 2021 these issues did not receive any particular attention.

External stakeholders

The main areas of discussion at the second consortium meeting were how to meaningfully involve external food system stakeholders in knowledge creation and how the project could have positive impacts on the food system. One main concern raised was about engaging different stakeholder groups in the educational case studies, given that actors have different aims and expectations and often people speak different ‘languages’. The aim must be that everybody involved in transforming education should learn and derive something new from participating. To some consortium members it is still not always clear what role external stakeholders play and how they contribute.

### Network learning outcomes

According to the concept of network learning developed by Knight & Pye (2005), the content of network learning may include observed key changes to network-level interpretations, practices and structures occurring within a given period of time. For the NEXTFOOD consortium the achieved learning outcomes were identified in workshops at the annual conferences and are listed in Table 3.

**Table 3.** The NEXTFOOD consortium learning outcomes.

Content of network learning	Observed learning outcomes
Changes to network interpretations	• Increased understanding of overall project structure • Stronger ties between members of the network • Increased understanding of key concepts, such as new educational approaches
Changes to network structures	• Innovative educational cases started at partner universities • Educational approach disseminated to an extended network • In-the-field stakeholders were connected to educational cases
Changes to network practices	• Introduction of action-oriented and student-centered learning • Establishment of multistakeholder learning arenas • Implementation of a new set of skills for agrifood and forestry professionals • Collaboration in an on-line setting

Changes to network interpretations

The consortium members were offered opportunities for discussing and reflect together in participatory workshops, as described in the methods section. As a result of participation in these activities they gradually developed a common understanding of the project’s overall structure and its key concepts. The initial frustration experienced by consortium members in not fully seeing the whole picture decreased by the end of the wrap-up session at the kick-off meeting. Consortium members said they had gained an improved understanding of the educational approach used in the project, both in the case development process as well as in the action research process. The consortium conferences in 2019 and 2020 offered an overview of the network, and contributed to an increased understanding of the different project components and how they were interrelated. One workshop outcome was that consortium conferences contributed to strengthening the ties between consortium members, which they consider crucial for good collaboration during the course of the project. This was reflected in a workshop note when participants were asked to reflect on achievements during the conference: ‘We got a common feeling for project spirit, first steps for a shared understanding and methodology’ (Consortium conference in Malmö, Sweden, 2018. Wrap-up session).

Changes to network structures

Changes to network structures relate to how network learning outcomes were conducive for changing existing educational programs and courses, as well as starting new ones, at the partnering institutions. These changes also include how new constellations of academics and external stakeholders were formed. New pilot-courses initiated at some of the partnering institutions and existing courses that were subject to improvement ( https://www.nextfood-project.eu/case-studies/) implied a long-term commitment by institutions, teachers, and students. The fact that most pilot courses were successful and took steps toward becoming institutionalized demonstrated how learning outcomes led to structural changes in the network. The educational pilots gained increased attraction among students and institutions. For example, in one partner university a short course in agroecology was developed into a full MSc program that started during the third year of the project (Master in Agroecology and Food Soveregnity at the University of Gastronomic Sciences of Pollenzo, Italy, https://www.unisg.it/corsi-iscrizioni/master-agroecology-food-sovereignty/). A new Agroecology program started at University of Chile with a first batch of students in 2021. Two courses covering social sciences, business modelling and agri-entrepreneurship started at Sekem foundation and Heliopolis University in Egypt and at Calcutta University in India. A new course focusing on biodiversity and nature considerations for forestry professionals started at Skogforsk, the Forestry Research Institute of Sweden ( https://www.skogforsk.se/english/projects/nextfood/).

Changes to network practices

Changes to network practices are the outcomes of a process where knowledge is shared and problems are solved in collaboration among network members. The outcomes of the network learning process were observed in how education was organized and operationalized at partner universities. Teaching practitioners strengthened their roles as facilitators, collegial teachers’ teams developed to support the implementation of the new educational strategies, and the teaching institutions more or less adapted to some degree to a new way of teaching. Learning arenas were established where teacher teams and students collaborated with external stakeholders in the learning process. Some partners disseminated the NEXTFOOD educational model beyond the consortium by teaching faculty and staff at other institutes by means of the methods developed, which is one indication that out-scaling was taking place. The permeating changes to the way learners were educated are seen as one consequence of fundamental changes in network practices. These include new competences for both teachers and students, for example how to perform and how to participate in a reflection session, and the shared meanings of learning situations that involve new practices. One example is understanding why and how reflection is an important competence and should be a part of the curricula. The travel restrictions in the second part of the project forced consortium members to develop new practices for virtual collaboration. For example, the consortium members self-organized on-line peer-learning groups for knowledge exchange and co-learning, which improved communication across tasks and work packages.

Network learning process

One year into the project, consortium members noted that the global network and the learning process works well, which indicates that the consortium had developed some qualities of a learning network. At a workshop about the learning culture of the project, held in between two annual consortium conferences in Vienna 2019, the 24 participating consortium members described this culture as open and supportive of participation and co-learning. Moreover, they said the culture was open-minded and that partners are open to try out new things. Members also highlighted a number of barriers that were impeding learning within the network. For example, they thought that internal communication sometimes was failing, e.g., there was a lack of feedback from project partners and consortium members had a narrow focus on their specific task which caused fragmentation. Another barrier to learning was that the educational cases had different characteristics and members struggled with challenges unique to each specific case, raising the need for more cross-case communication. The purpose of the workshop in Vienna was to stimulate co-learning among network members on the challenge in shifting from being a teacher to a learning facilitator, on action research in education, and to establish a productive collaboration among different elements of the project. These success factors had previously been identified at the consortium conference in Budweis, Czech Republic, 2019. 

The network learning process is about developing interorganizational knowledge, that is, “a common repertoire of experiences and know-how from which the participants can draw” ( Mariotti, 2012). This involves more than merely sharing existing knowledge, rather it involves participation in fruitful interaction with other members for co-learning and co-creation of new knowledge. In NEXTFOOD, the facilitators of the learning events aimed to combine dissemination of mid-term results and follow-up on milestones and deliverables through a process of dialogue, reflection, and visioning. This was a new approach to some network members, and some expressed frustration that too much time was spent on the process part and too little on formal project content (milestones, reports, deadlines). This is a parallel situation to what was experienced in many of the educational cases in the project, where some students struggle with the shift from passive to active learning in a diversity of learning arenas. Like these students, most consortium members soon came to appreciate consortium workshops for their stimulating and productive dialogues, leading to a shared understanding of project aims and stimulating collaboration. As facilitators of the network learning process we learned to establish balance between content and process at consortium meetings, and stressed the importance for members to be well prepared and well aware of the intended learning outcomes to be able to make a contribution and learn from the interactive sessions. In addition to the annual consortium conferences a series of workshops was organized on-line and in real life with the purpose of building capacity among the consortium members to work effectively for a transformation at educational institutions as well as in the consortium.

## Discussion

By a recent declaration, the European Education Arena 2025, EU-leaders aim to build an inclusive and high-quality education as a part of the green deal strategy ( European Union, 2020). Education that is transdisciplinary, learner-centered and challenge-based is particularly mentioned as an important means to foster ‘transversal skills’. Although policymakers and governments engage in the sustainability education agenda through top-down policy measures, the responses to educational policies are too often not translated into practical activities on campuses. Current literature tends to focus on niche-scale university initiatives for sustainability education that provide insights on various impactful teaching and learning approaches from a variety of different subjects. But empirical research is missing on the communication and learning associated with inter-organisational and international collaborations in higher education. It is important that teaching institutions share their achievements within the field of sustainability education with the global community, and research should evaluate mechanisms and potential venues for sharing experience of local scale among niche-level actors. The model presented in this paper could facilitate co-learning and collaboration by connecting innovative university initiatives into a supra-community and, in the long term support, a transition of the educational system within agriculture, foods, and forestry. 

In this paper, we used the concept of network learning to explore to what extent and how a facilitated consortium learning process can improve the capacity to change the educational practices in agricultural and forestry education, and thereby contribute to a transition of the larger educational system. In the context of learning for sustainability transitions, reflexivity is an essential process of introspective examination of one’s personal beliefs, values, and judgments which may impact the research process. This is seen as a vital quality that allows each person to challenge dominant regimes and methods by stimulating inquiry, dialogue and co-learning ( Beers & van Mierlo, 2017). We particularly consider the important potential role of the consortium in promoting sharing of ideas, successes, and failures. Among wider expected modifications in strategy are transformational changes from entire focus on content to a growing focus on process, while not abandoning the importance of essential content in courses and workshops. Another important change is away from traditional management and control to more inclusive cultures of education practiced and promoted by all participants in the learning landscape. A needed transformation is away from the current focus on isolated educational projects and their independent spheres of activities, towards a system that will give way to some extent to an efficiently functioning collaborative network of projects. This does not suggest that all projects will follow the same menu of educational methods, but rather will pursue the most appropriate strategies to meet their goals in each particular ‘educational terroir’ *.* An essential component includes communication within the network about what each group is doing and encouraging a sharing of their evaluations of lesson learned including successes. This will result in a larger ‘learning network’ that will base future planning on local successes as well as ideas from other network participants, and eventually from other networks.

We recognize that the development of educational projects is influenced by specific contextual factors of the region in which they have been carried out. Despite the individual differences specific to the location, the projects struggled with similar challenges in gaining acceptance for the new educational approach among teachers, students and managers at their home organisations. These challenges were identified on multiple occasions in the workshops of the consortium and are mentioned in a summary list of challenges above. Most case leaders were positive about the benefits they experienced as part of the collaborative network. They felt that the network provided necessary knowledge and support while they were implementing the new educational approach. They also mentioned gaining an improved image from being associated with the network was helpful in achieving acceptance for the initiative among colleagues and managers. 

The network learning process helped the consortium overcome challenges and implement a new learning model at the partnering institutions, i.e. structural change happened despite frictions between the educational innovation and the institutional context. This is in line with Beers & Van Mierlo (2017) who described a situation where an increased reflexivity leads to a considerable influence on the institutional setting, hence paving the way for change. Although change happened in all educational case studies, the road to get there was different for each of the partners. Most importantly they started at different levels of pre-knowledge and operated in vastly different contexts, which influenced the direction and the pace of moving forward. 

The UNESCO report “education for sustainable development goals” ( UNESCO, 2017) highlights the importance of developing systems thinking through action learning to be able to deal with socio-ecological complexities, such as the agrifood system. To develop a holistic view is challenging not only to students but also to academic staff, as reflected in the consortium’s struggle to take an integrated approach on the project. Even though consortium members saw the possibility of creating synergies by closer collaboration, they expressed a lack of understanding of the work done by other project teams and could not always see how to go about collaborating in practice. To avoid compartmentalization, they expressed a need for clear direction and for help to link their activities to other parts of the project, which became more difficult when the pandemic hit and physical meetings were restricted. The consortium members viewed NEXTFOOD as a network organization, connecting different disciplines and a wide diversity of stakeholders. On one hand this generated dynamic and creative interaction, but also resulted in a somewhat loose project structure, unclear goals and roles, which members sometimes found confusing. To overcome such vagueness, Ayre *et al.* (2018) suggested that individual roles and processes for working together should be negotiated in the initial phases of a co-creative research initiative. Scott *et al.* (2018) argues that new tools are needed in the management of collaborative project networks with emergent dynamic properties. An implication of our experience is that an effective coordination instrument has to be in place from the beginning to promote communication across partner organizations. Collaboration in networks leaves more responsibility to the participants in following up their work, autonomously taking individual initiatives to collaborate within the network, and keeping updated with other parts of the project. According to Oerlemans & Assouline (2004), a successful management of innovative networks means to combine leadership with shared responsibility and commitment. Ollus *et al.* (2011) conclude that in collaborative project management, focus is on understanding and monitoring tangible assets like communication, collaboration performance, and trust. Decisions are made in a decentralized and democratic manner, which implies that partners share a common understanding of project objectives, requirements and practices, and then have sufficient skills and competences to accomplish their project tasks.

In NEXTFOOD, partners sometimes had a narrow focus on completing their own responsibilities in the project at the expense of looking to the whole project. It is essential to design a reward system, through shared publication of journal articles or special recognition of each partner project, that will provide the needed incentives for each participant not only within their own organization but to see the value of engaging with and learning from others. Partners expressed that in order to improve collaboration it would be necessary to develop further a common understanding of purpose, aims and fundamental concepts of the project. It became clear that partners of the consortium interpreted concepts central to the project differently, which hampered project development because communication and collaboration were more difficult without shared meaning. For example, ‘cyclical learning’, ‘sustainability’, and ‘skills’ had different meanings to different partners. This is not surprising when the language of the project is English, which is not the native language for most participants, and there are multiple definitions of terms used in the literature and in educational improvement programs. In relation to this discussion, it is valuable to mention here the difficulties the consortium members experienced with remote collaboration. Developing collaborative networks is easier when meeting face to face, which allows communication with people you haven’t met in person before, to establish more personal relationships that allow people to be more direct and informal. It requires building mutual trust to establish strong networks of institutions and foster frank and honest exchange among participants.

In the field of sustainable management of natural resources, there is an increased understanding of the importance of involving different sets of actors with complementary types of knowledge throughout the whole research process, from initial planning to dissemination and demonstration ( EU SCAR AKIS, 2019). The external stakeholders were involved to various degrees in the different projects. In some cases they were mentoring the student teams in the product development process, while in other cases they gave input more from an external perspective. Engaging external stakeholders in a broad sense helps expand their roles from providing a farm to visit, an agricultural or forest-related business to analyse, an input supplier to evaluate, or other farming, forest, or food system activity where students can conduct interviews to learn about their goals, activities, and challenges. All these are important, yet they put the stakeholders primarily in a ‘teaching mode’ as suppliers of information. It is often a new challenge for some to be invited into the planning and evaluation cycle of the total learning process, and may not be one to which they are willing to dedicate additional time and energy. Individual projects tended to seek stakeholders who would be best suited to participate in such a mutual learning process. Facilitating a learning process is common to essentially all teaching programs, but to truly engage students and stakeholders as co-learners we need to add the responsibility to contribute to design of the sequence of topics and practical activities in a course and the activities needed to enhance learning.

Although it was anticipated that serious participation in the network would lead to positive pay-offs for each project, this means that members would justify the time and energy investment in the network in terms of advancing their own group’s goals. It is important that those leading the NEXTFOOD network recognize and respect the fact that each group has the obligation to assess the costs and benefits for them to continue to participate in the network, or whether their goals would be better met by using what they have learned to develop autonomous and independent activities. This is in keeping with the principles of uniqueness of *terroir* and the need for using scarce resources to best meet individual group goals. It is a strength of the network to realize that some groups may choose to ‘do their own thing’ if they do not recognize advantages of working in a network.

## Conclusions

The consortium action learning process connecting several multistakeholder research activities in a supra-community that contributed to network-level learning outcomes. This was observed as changes in how network members interpreted the new educational approach, the widespread implementation of new educational practices at teaching institutions, and the structural changes at the partnering institutions. These results were achieved also in cases where the institutional environment was far from supportive. Based on experiences of the consortium members, we have learned about successes as well as difficulties during implementation of the transformational process. Management of an inter-organisational collaborative network must early in the project clearly communicate the project structure with roles and links between different tasks and work packages to help partners in finding their place in the network and identifying important key synergies.

The responsibility of each individual partner must also be clearly communicated. There should be a constant learning process and ample opportunities to discuss and reflect together in participatory workshops, and these should be offered early in the network activitiers. It is important that meeting facilitators try to balance project content with an effective learning process. Even though this initially may evoke some resistance it will contribute to developing a shared understanding of important terms and key concepts. To foster network learning, the achievements of annual consortium conferences must be followed-up by collaborative activities in between these meetings. 

To adapt to new realities such as the pandemic and the goal to reduce climate impact, the consortium collaborated in a virtual environment during much of 2020 and 2021, and learned that routine tasks such as project follow-up and presentations in one-way communication mode can preferably be done remotely while more complex processes involving more people is better done in face-to-face meetings.

Lessons learned in NEXTFOOD will reveal methods that are most useful to facilitate the transformation needed by individual teachers, by students, by administrators, and by stakeholders. Progress so far with a wide range of learning activities in different educational organizations has been encouraging, and we look forward to seeking continuing grant support from EU or elsewhere to support projects that will contribute to a wide transformation in participatory education.

## Data availability

### Underlying data

Zenodo: Educational transformation and network learning dataset – qualitative data from an international collaborative EU-project. DOI: https://doi.org/10.5281/zenodo.5810106. ( Melin *et al.,* 2021).

This project contains the following underlying data:

- SLU_V1.0_Consortium Workshop Notes_2021.12.29.docx. (The file contains the compiled notes from the workshops organized at the four annual consortium conferences; Malmö, Sweden 2018; Budweis, Czech Republic 2019; On-line 2020; On-line 2021).

Data are available under the terms of the Creative Commons Attribution 4.0 International license (CC-BY 4.0).
