# Facilitating transformative innovations in sustainability education

**DOI:** 10.12688/openreseurope.14407.2

**Published:** 2022-06-15

**Authors:** Martin Melin, Geir Lieblein, Tor Arvid Breland, Charles Francis

**Affiliations:** 1 Department of People and Society, Swedish University of Agricultural Sciences, Box 190, 234 22 Lomma, Sweden; 2 Department of Plant Science, Faculty of Biosciences, Norwegian University of Life Sciences, P.O Box 5003, 1430, Ås, Norway; 3 Department of Agronomy and Horticulture, Institute of Agriculture and Natural Resources, 279 Plant Science Building, University of Nebraska –Lincoln, UNL, Lincoln, Nebraska, 68583-0910, USA

**Keywords:** Action learning, education, systems thinking, educational transformation, agrifood system

## Abstract

Educational strategies globally are changing from an authoritative, top-down model to one focused on greater student and stakeholder participation in planning and implementation of research and educational activities. In addition to emphasis on student-centered education, strategies currently evolve to encompass learning organizations and multistakeholder learning networks. These are essential to address the complexity and scope of tomorrow’s challenges, involving issues that could be called ’wicked problems’ not easily addressed by single disciplines nor resulting in solutions that please all the players. In this study we describe how a transformative innovation – the NEXTFOOD educational approach – may contribute substantially to a transition of agricultural and food education and how it can be developed and diffused within and between teaching institutions. The method was action research informed by several workshops organized at annual consortium conferences during the first three years of the project. The findings show that a successful transformation involves learning both within and across innovation projects repeated at various organisations in a network. The action research model presented in this paper may be useful as an instrument to support the facilitation of transformative innovations. The transition process resulted in substantial changes in mindset, educational practices and organisational structures at the teaching institutions. However, scaling-up promising educational initiatives may encounter several barriers that need to be overcome at individual, group and institutional levels, and we provide insight on how this can be accomplished in a multi-national consortium of universities.

## Plain language summary

The sustainability challenges of agriculture and food production, such as accelerating climate change and the immense loss of biodiversity, are the kind that cannot be adequately addressed by single disciplines and cannot be solved to the satisfaction of all the players. Dealing with such problems will require learning approaches that build on many sources of knowledge, as well as adopting proven practices learned from farmers and other practitioners with long experience.

NextFood is a twelve-country collaborative network that aims at fundamental transitions towards more sustainable food production, by bringing together university students, academics, field professionals, farmers and other stakeholders in order to create a community of learning from experience and research. In addition to introducing new teaching methods into their own courses and programs, innovative teachers met regularly during the course of the EU Project to share experiences so that the collaborative network itself becomes a learning community. The question this research study set out to answer is how a new approach to sustainability learning can be developed and scaled-up in order to contribute to a fundamental change in the way education is done at universities today. By testing this new educational approach in pilot projects at different universities and sharing that experience in a network of teaching institutions, teachers grew by shifting their mindsets on ‘what is good education’ and began to test and accept new practices in their teaching. A number of barriers to the implementation of the new approach were identified that should be addressed in future initiatives.

## Introduction

Human impact on earth has accelerated to unprecedented levels, and we now see the consequences of an accelerating loss of biodiversity and other massive impacts due to continuing climate change. Human survival faces a risk from exceeding the catastrophic tipping points of planetary systems, which would threaten current societies and ultimately life on earth ( Rockström *et al.,* 2009). The expansion of land used for production of food plus use of high-input modern farming practices are among current key drivers in the destruction of natural ecosystems, loss of biodiversity as well as climate change ( IPCC, 2019). Objectives of the European Green Deal are to make Europe the first climate-neutral continent by 2050 through adopting an economic growth strategy that is fair and green. To achieve this ambitious goal will require a system-wide transformation that steers all sectors of society away from the current paths of mass production and mass consumption, carbon-use intensity, and heavy reliance on non-renewable natural resources ( Scot & Steinmuller, 2018). Higher education is one key that can help us achieve the European Green Deal and the Sustainable Development Goals; sustainable development should be incorporated in the curricula across all disciplines and levels of the educational system ( European Union, 2020). Socio-technical regimes, such as the educational system, consist of networks of actors, including academic staff, specialists from industry, students, public authorities, researchers, and those in financial institutions who through their coordinated actions most often contribute to the continuation of the mainstream way of doing things ( Geels, 2002). Calling for a new mode of sustainability education means challenging assumptions and ideas of ‘good educational practices’ engrained by experience and tradition from students, faculty and members of university management. In transition theory, experimentation in socio-technical niches plays an important role as a driver of change.

Such transition experiments are small scale innovation projects addressing persistent problems in the sector where the expected outcomes are radical changes in culture, structure and practice ( Loorbach & Rotmans, 2010). Recent empirical work has put the focus on “transformative innovations”, that are initiatives emerging on a local level driven by engaged citizens, entrepreneurs, or other actors in response to pressing sustainability challenges ( Loorbach *et al.*, 2020). In these kinds of innovation, the technology element is typically less dominant, and instead they are focused on finding new ways of providing basic societal needs such as food, energy and education. Although they are rooted in the local context, they are also part of a translocal network of similar initiatives that are sharing ideas, knowledge and experiences (ibid.). The focus of this study is a shared transformative innovation – the NEXTFOOD educational approach -- that was collectively developed by several teaching institutions including shared meanings and practices on action-oriented sustainability learning.

In this paper we explore and describe how such an innovation may contribute substantially to a transition of agricultural and food education and how it develops and diffuses among teaching institutions. An understanding of the mechanisms behind this dynamic process opens the possibility to manage transitions of educational systems towards sustainability action learning, or at least give them a nudge in the right direction. The research outcomes are based on action research in twelve case studies that involved the implementation of transdisciplinary learning approaches in practice, in the context of courses and programs related to food, agriculture and forestry, covering a wide geographical area and different levels of education. In the case studies, multiple stakeholders participated in a process of co-learning to find solutions on local sustainability problems and to improve the educational methods. Cases were linked by creating a learning arena that provided a foundation for impacting the wider educational sector through organizing a broad participatory co-learning process.

In the following section we build a connection between key literature on transition studies and education for sustainable development with a particular focus on the mechanisms that convey system transitions. Then we present the action research model used in the NextFOOD project to enact learning on a local through global scale and the methods used for the empirical analysis in this study. In the results section the changes in mindset, educational practices, structures and identified barriers to change are presented with examples from several case studies. In the analysis and discussion we draw on the results of the case studies to derive conclusions on how facilitation of transformative innovations in education can be improved.

### Transition of the educational system

In an age of accelerating change where society seeks to develop pathways towards a more sustainable future, there is an increased recognition of the need for parallel and innovative educational responses ( Stephens *et al.,* 2008). Inflexible educational and research institutions locked into outdated traditions and practices will not be equipped to address the complex sustainability challenges our societies are facing ( Cortese, 2003). Sustainability challenges require an alternative educational approach that is action-oriented, transformative and supports self-directed learning, participation and collaboration ( Lieblein *et al.,* 2019). Promising educational programs informed by agroecology ( Francis *et al.,* 2017; Rivera-Ferre *et al.,* 2021), where learning about multifaceted issues about the food system by working closely with farmers and food system stakeholders, are gaining interest with educators and students in both formal and non-formal settings around the world. Even though there may be good arguments for a shift in education, often development and change are locked-in by the organisation of education into strictly separated disciplines and dominated by educational activities that have become disconnected from society. This effectively hinders the integration of knowledge from different fields and strengthens the tendency of universities to focus on generating theoretical and abstract knowledge rather than new and proactive strategies that can be applied to real-world problems ( Evans, 2015). The lack of an interdisciplinary approach constitutes a key barrier to reforming the agri-food system ( Valley *et al.,* 2017), and risks undermining the capacity of those in the professional workforce to cope with sustainability challenges in holistic and creative ways ( Coops *et al.,* 2015).

As transdisciplinary researchers engaging in an international network of teaching practitioners working to co-create a future roadmap for transformative education in agriculture, foods and forestry we were interested in understanding how innovative educational approaches developed, became institutionalized and spread beyond the project consortium itself as a starting point for generating knowledge on transition management. This paper builds upon extensive empirical work in the NEXTFOOD project where 12 in-depth case studies in education were conducted, mainly relying on workshop outcomes collected at several consortium conferences organized during the four years of the project. In order to understand the dynamic mechanisms behind development and diffusion of case studies, and how they impact (or not) the incumbent mainstream educational and agricultural practices, we draw upon the typology described by Van den Bosch (2010) that includes ‘deepening’, ‘broadening’ and ‘scaling-up’.

*Deepening* is a mechanism where actors together learn from the experience of trying to collaboratively enact change within a specific context. It is associated with developing shared meaning among actors from different backgrounds and practices. This is a useful process in transition experiments where stakeholders are organized in learning arenas to resolve differences in meanings and to co-create solutions that fulfil societal needs in a different way ( Loorbach & Rotmans, 2010). In agri-food education, pioneering teaching practitioners leading local transdisciplinary education initiatives may contribute substantially to sustainability transition by engaging in processes of deepening with other groups of teachers practicing similar educational strategies.

The mechanism *broadening* is according to transition theory a key mechanism by which experiments in multiple contexts collectively contribute to an emerging change in culture, practice, and structure ( Van den Bosch & Rotmans, 2008). According to Van Den Bosch (2010) there exist two types of outcomes of a broadening process: either a new deviant idea or way of doing things gets diffused or adopted in a variety of contexts, or innovative methods fulfil a broader societal function. For example, broadening within the agricultural educational sector would entail a shift in mindset about what sustainability education should be, and new practices or structures in education would get diffused within a certain context (i.e. within food and farming education) or into new sectors or groups of learners beyond agriculture. *Scaling-up* a transition experiment means that an innovation sticks to the dominant regime and influences the mainstream way of thinking (mindset), doing (practices) and organizing (structures) at the level of a societal system (ibid.). The results of scaling-up in education are fundamental shifts in the dominant ways education are pursued, which go well beyond the scale of the initial transition experiment.

### Background and structure of the NEXTFOOD project

As a response to the need for innovation in transition education, a 12-country intitative funded by EU through the Horizon 2020 program, our NEXTFOOD (NF) project ( https://www.nextfood-project.eu/) was established to bring together teaching practitioners from several countries to co-create a future roadmap for education in agriculture and foods. Network members are researchers and teaching practitioners from diverse disciplines such as agroecology, social sciences, food studies and farming systems, as well as representatives from non-governmental organizations (NGOs) and business networks. Case-studies of educational initiatives designed for action-oriented education, sharing of experiences and research, are primary activities of the network. The NF educational approach developed in the case studies was expected to support students to bridge the gap between knowledge and action, and to enable responsible action in the wider sustainability context. It was built on literature in the area of sustainability education, such as the UNESCO report “education for sustainable development goals” ( UNESCO, 2017), which highlights the importance of developing systems thinking through action learning to be able to deal with socio-ecological complexities. A learning arena was established on the network level, focusing on testing new strategies, evaluating their successes, and providing a foundation for impacting the wider educational sector through organizing a broad participatory co-learning process.

Partnering organisations are Swedish University of Agricultural Sciences, Lund University and Skogforsk, Sweden; University of Oradea, Romania; University of South Bohemia České Budějovice and Bioinstitut, Czech Republic; Norwegian University of Life Sciences, Norway; American Farm School, Agronutritional Consortium of the Region of Central Macedonia and International Hellenic University, Greece; University of Bologna, International Center for Advanced Mediterranean Agronomic Studies and University of Gastronomic Sciences of Pollenzo, Italy; Deutsche Welthungerhilfe, Germany; Sekem Development Foundation, Egypt; Mekelle University, Ethiopia; ISEKI-Food Association, Vienna; Roskilde University, Denmark; and University of Chile, Chile. Network members are researchers and teaching practitioners from a diversity of disciplines such as agroecology, social sciences, food studies , farming systems, and forestry, as well as representatives from NGOs and business networks. On the network level, a learning arena was established with focus on testing new strategies, evaluating their successes, and providing a foundation for impacting the wider educational sector. Within this arena, the facilitation of a broad, participatory, action-oriented co-learning process was facilitated to ensure that research outcomes and practical experiences gained within working groups of educational pilot projects, then were circulated between different project domains. One goal was to promote institutionalizing transformation within the partnering organisations.

**Figure 1.**
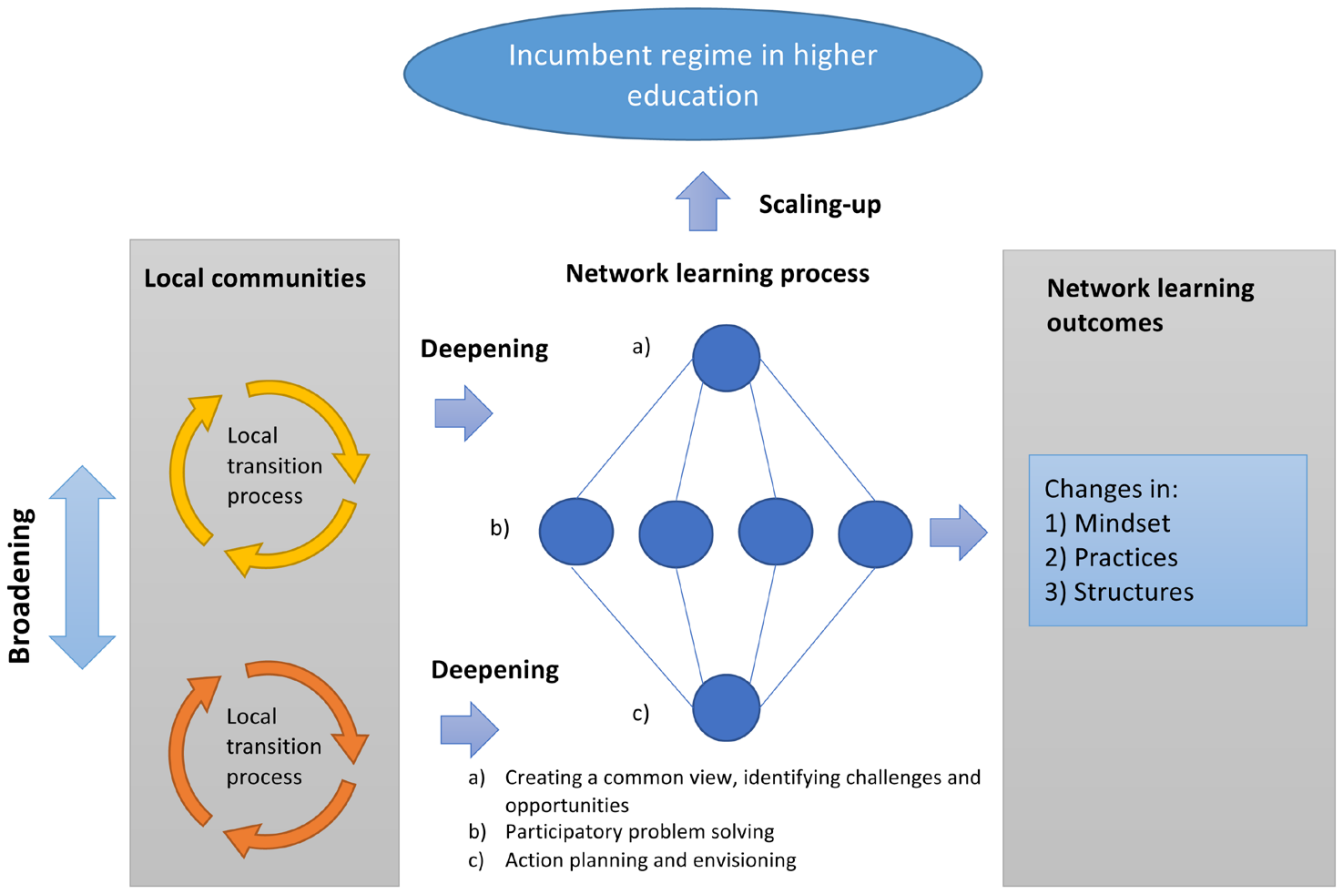
The consortium members engaged in a process governed by the NEXTFOOD action research model. In this model, a three-step consortium action learning cycle was linked to several multistakeholder research activities and local learning arenas run in parallel.

At annual consortium conferences, 4 in total, members of the consortium had opportunities to engage in a process of problem structuring, action planning, and co-learning. Educators in each educational case form learning communities, and the consortium becomes a supra-community with all the players contributing their experiences and lessons learned ( Figure 1). The workshop design at these meetings was similar each year. It was planned as a learning cycle, starting with collective generation of an overall view of the project, then going into more detailed discussions about project matters on team and task level, and finally assembling the pieces into an action plan for the coming year. By engaging in dialogue, consortium members together developed an agreed-upon meaning of aims, educational approach and the overall project vision ( *deepening*). With the support from the more experienced case leaders, the NEXTFOOD educational approach was adopted by all the twelve cases in a peer-learning process and spread within the teaching institutions ( *broadening*). Outcomes of the network learning process provided the basis for a strategic roadmap and step-wise guides for any actor outside the consortium who wants to introduce action learning in their courses or programs ( *scaling-up*). 

## Methods

### Ethics statement

The research has been conducted under the ethics requirements and guidelines of the NextFood project (Deliverables 8.1, 8.2 and 8.3), which all comply with regulation (EU) no. 1291/2013 of the European Parliament and of the Council. A data management plan was developed to ensure that data collection and processing was performed in accordance to the GDPR. Written informed consent for publication of qualitative research data was obtained from the consortium members prior to participation.

### Approach

The action research approach described here was informed by several workshops organized at annual consortium conferences and at two additional meetings for case development during the first three years of the project (May 2018-May 2021). Action research is a cyclical process where knowledge generation is combined with an active engagement of researchers in solving complex societal problems ( Levin & Ravn, 2007). In addition, our analyses build on observations made at meetings organized for consortium members in between the annual conferences. The workshops were facilitated by researchers in the NF project. The first workshop was organized in Malmö, Sweden in May 2018 to generate a mutual understanding of baseline conditions. Qualitative data were collected including viewpoints of faculty members and teaching professionals about project aims, ambitions, and consortium organization as well as barriers and opportunities for implementing research tasks of the project. The same assessment was done at three following conferences, in Budweis, Czech Republic in June 2019, and two conferences organized online in June 2020 and in May 2021. At the on-line conferences we used the Zoom meeting platform.

At the annual consortium conferences the members were asked to individually reflect, and then discuss in groups and in plenary a) what is most challenging to the development and implementation of new action-oriented educational approaches in higher education, b) what has been achieved since last the meeting, c) what was achieved during the meeting, and d) implications for the future. At two additional meetings, in Pollenzo, Italy in 2018 and in Vienna, Austria in 2019, the majority of consortium members participated in workshops focused on knowledge sharing between educational cases and discussed the progress of each case.An overview of the meetings used for empirical data collection is given in Table 1.

**Table 1.** Location, date and total number of participants of the six consortium conferences.

Location	Date	Number of participants
Malmö, Sweden	6 May — 4 May, 2018	48
Pollenzo, Italy	17 Sept —19 Sept, 2018	28
Budweis, Czech Republic	28 May — 30 May, 2019	47
Vienna, Austria	23 Oct — 25 Oct, 2019	24
On-line	3 June — 5 June, 2020	80
On-line *	4 May — 25 May, 2021	26

*The activities and meetings of this conference were spread out during three weeks. Number of participants based on participants at the catch-up session only.

## Results

In the following sections we present how implementation of the educational approach changed the cases in terms of mindset, practices and structures (summarized in Table 2), and thereby contributed to a transformation of the educational system. In the last section we clarify these by providing some examples from the cases.

**Table 2.** Changes in mindset, educational practices and structure observed in the 12 local cases.

*Mindset:* ○ Teachers are embracing a holistic view on education ○ Introduction of a real-life field case is used as a starting point for the learning process ○ An understanding is gained for the new roles of teachers in action-oriented education
*Practices:* ○ Action-oriented activities are introduced in courses and programs ○ A shift to more learning arenas outside university campus is implemented ○ Learners are educated in core sustainability competences
*Structure:* ○ Teachers take the role as learning facilitators ○ Teacher teams are formed to implement the new learning approach ○ New action-oriented agroecology programs start at partnering universities

### A shift in mindset; going from lecturing in strict disciplines to facilitating holistic education

The NextFood case leaders described the transformation of education from being strictly separated into topics and disciplines into one that could support students to integrate different sources of knowledge and develop systems thinking. For example, one case offered three master programs in integrated pest management, water management and Mediterranean organic agriculture. The course plans are organised in a traditional way with subject-specific courses, with an individual project during the first year and thesis-writing in the second year. There is a clear distinction between the different subjects, as well as between years. The case aimed at transforming the master program in organic agriculture to become more holistic, where courses and teaching activities were more closely integrated and performed in collaboration with extra-university stakeholders. Another example is a case that educates the future gastronomist, i.e. “a man or a woman capable of thinking and framing food critically, as one of the crucial aspects of understanding the world and also fundamental for its transformation” (case leader, Pollenzo 2018). For the gastronomist the food includes: “objects of knowledge in its many aspects” and “ a means of interpreting reality as a whole” (ibid.). These two examples show how the NextFood case leaders aim at training learners in taking a systemic approach to solve the complex challenges in the farming and food system. Moving students to the field as a starting point for their learning journey is very different from the more traditional pedagogy at universities and challenges the academic culture where theories and conceptual knowledge is in focus. By gaining field experience students get an understanding of the farming system and how practical experience can be connected to theoretical knowledge. The case leaders quickly appreciate how action-based learning contributes to a sustainability transition on many levels -- by students aquiring sustainability skills, by the interaction of academia with the wider community, by contributing to the development of a generic curricula and by spreading of the action-oriented approach beyond the own institution. After changing to an action-based learning model “the students and me realized that it is high time to come out of the classrooms and apply those ideas in society and thus become agents of change” (case leader, Pollenzo 2018). Case leaders say that making this transformation is not always easy and requires a shift of mindset among teaching peers and students. In a discussion at the Vienna workshop in 2019, the case leaders agreed that teachers need training in facilitation as well as in the core competences. The consortium members concluded that the mindset of a good facilitator is someone who is *well prepared* in order to be flexible and in control of the schedule; *inclusive and patient*, making sure everyone is heard; *ethical*, accepting other views than your own; and *aware* of the group process and culture.

### A shift in educational practice to producing knowledge that matters

The purpose of action-oriented education is not only to educate students but also to have an impact on extra-university stakeholders, and thereby have an impact on the local food system. All cases organized a multistakeholder platform consisting of academics, students and key stakeholders (local authorities, farmers, rural entrepreneurs, cooperatives and associations). In this local innovation system participants could act together in a way to find solutions and answering to specific demands. The aim was to create “knowledge that matters” (Case leader, Pollenzo 2018), where the knowledge creation process is driven by the needs of stakeholders. In an action research process where outputs from one course cycle are being used in the next version of the course and for feedback to the stakeholders, the intention was to combine knowledge development and the facilitation of change. In one example, the case leaders supported academic staff to go from teaching in their narrow topics to become a part of a multistakeholder platform. They identified a thematic or geographical area that could form a common ground for the multi-sectoral and multi-disciplinary teaching. They connected their case to a coastal park in southern Italy where agricultural activities and tourism co-existed with high ambition to protect nature, preserve biodiversity and cultural-historical heritage. In one of the course cycles, student teams worked together with food entrepreneurs to increase the valorization of organic food from the local area. In another case, a course focusing on forest biodiversity and nature considerations, forestry professionals used social media to share their insights and observations from the field in a community of practice consisting of other professionals and experts. This was an effective way to improve horizontal learning and to implement nature-friendly logging techniques.

The NF cases made an effort to mobilize key stakeholders, who are not seen as passive actors; instead they are supposed to take a very active role in education. The external stakeholders were involved to various degrees in the different projects. In some cases they were mentoring the student teams in the product development process, while in other cases they gave input more from an external perspective. One case involved a network of farmers connected to Slow Food communities in various countries. The farmers opened their farms for field visits and invited students to spend three months in their communities for doing action research. By building long-term relationships between faculty and stakeholders, students gain practical experience and the external partners gain access to faculty expertise and the creative capacity of student teams.

Another example on the change to education driven by societal needs is a NEXTFOOD partner that runs a food supply chain innovation competition game open to any team of master-students globally. A key change in the competition that was implemented during the NextFood project is going from a research question was defined by experts to questions are identified by the participants themselves. By working on real problems defined by students this education can contribute to pushing the green shift of farming and food systems. The winning teams of the past two years developed a solution for reversing food waste to probiootic food in Nigeria, and enhanced local food access in the state of Kentucky. “This competition also pushes the green shift to reality because the problem of climate change becomes a specific problem related to food that you can work on at your university and solve in a couple of months” (Case leader, Pollenzo 2018). One apparent change in all cases was an increased focus on soft skills in courses, such as collaboration and facilitation that teachers saw as important for students to acquire for being able to fully participate in action learning activities. These changes were reinforced by the positive feedback from students who embraced learning these new competences and appreciated the improvements in teaching methods.

### A shift in educational practices to a focus on learning processes and methods

For the cases, experiential learning became an integral part of education and the universities organised many study trips where students engage in experimental activities in laboratories, in the field and in school gardens. In addition, students are inspired to volunteer in the activities organized by farms networks as an extra-curricula activity. Even if some of the case leaders have been running an action-oriented short course for several years before the project, they emphasize the importance of improving their theoretical and practical knowledge on experiential learning. “Every year brings me something”, one case leader said to stress the continuous improvements between each course cycle (Pollenzo 2018). Case leaders stress that other teachers too should be more aware about the importance of learning processes and methods, and point out that they should reflect on course outcomes and strive to become what is called a *reflective practitioner*. The professors involved in many of the NF cases are coming from different disciplines and don’t always share a common view on the importance of learning processes. One case ran four different competitions in the four years of the NF project, and after each competition they try to improve education and training methods based on the feedback from participants. One example from this particular case was the development of interactive online workshops where the students were given the opportunity to train soft skills. Knowledge on experiential learning is also necessary for students, so that they are able to support the learning process and learn how to become involved in agrifood systems, how to reflect on their experiences, and build their knowledge on what they have learned. One year into the project, consortium members noted that the global network and the learning process worked well. At a workshop about the learning culture of the project, held in between two annual consortium conferences in Vienna 2019, the 24 participating consortium members described this culture as open and supportive of participation and co-learning. Members also highlighted a number of barriers that were impeding learning within the network. For example, they thought that internal communication sometimes was failing, e.g., there was a lack of feedback from project partners and consortium members had a narrow focus on their specific task which caused fragmentation.

Although reflection on experiences is fundamental to learning, such elements are not often a natural part of education today, and action-oriented learning methods are seen as the exception to a long-accepted conventional one-way style of teaching. This became obvious in one of the cases in India that runs a certificate course in agroecology with action-oriented learning. The teaching used to be entirely based on theory transferred by teachers lecturing in a monologues style. There was a total lack of transdisciplinary course content and for students the only purpose of education was merely achieving a degree and accumulating theoretical knowledge. In line with the motto of the university: *Karmani Vyajyathe Prajnja* meaning “Wisdom manifests itself in action”, the course transformed into a holistic action-based learning model and since then there has been a positive shift among learners in the development of the NF sustainability core competences (observation, reflection, dialogue, participation, envisioning, facilitation).

### Changes in structures through initiation of new programs and courses

Changes to network structures relate to how network learning outcomes were conducive for changing existing educational programs and courses, as well as starting new ones, at the partnering institutions. These changes also include how new constellations of academics and external stakeholders were formed. New pilot-courses initiated at some of the partnering institutions and existing courses that were subject to improvement ( https://www.nextfood-project.eu/case-studies/)

implied a long-term commitment by institutions, teachers, and students. The fact that most pilot course leaders were successful and took steps toward institutionalizing their new activities demonstrated how learning outcomes led to structural changes in the network. The educational pilots gained increased attention and attraction among students and institutions. For example, in one partner university a short course in agroecology was developed into a full MSc program that started during the third year of the project (Master in Agroecology and Food Sovereignity at the University of Gastronomic Sciences of Pollenzo, Italy, https://www.unisg.it/corsi-iscrizioni/master-agroecology-food-sovereignty/). A new Agroecology program started at University of Chile with a first batch of students in 2021. Two courses covering social sciences, business modelling and agri-entrepreneurship started at Sekem foundation and Heliopolis Univeristy in Egypt and at Calcutta University in India. A new course focusing on biodiversity and nature considerations for forestry professionals started at Skogforsk, the Forestry Research Institute of Sweden ( https://www.skogforsk.se/english/projects/nextfood/).

Another structural change is related to the new role of teachers as learning facilitators that was gradually accepted by the teachers at the partnering institutions. One year into the project most of the partners were convinced about the benefits of the new learning models. Our observations and conversations with consortium participants and other educators in our universities is that this transition is well under way, and more people every year are ‘buying into the concept’ of co-learning and accepting the reality of depending on a wide range of information resources beyond the personal expertise of teachers. However, at the consortium meetings the consortium members pointed out several barriers to the wider implementation of the new educational approach, which are presented in the following section.

### Barriers

At the annual consortium conferences the members were asked to individually reflect, and then discuss in groups and in plenum “what is most challenging to the development and implementation of new action-oriented educational approaches in higher education”. The outcomes of this discussion are reported in this section as barriers on the individual, group and institutional levels.

*Individual level*

Motivation of students. There was concern that all students may not be ready for this transformation and it is necessary to understand their attitudes toward their own learning. How to support student motivation and engagement in the new learning landscape came up in several workshop notes. Although most students appreciate action-oriented activities, differences in their backgrounds and pre-knowledge levels influence how well prepared they are for self-direction in the learning process. 

Teachers becoming learning facilitators. A concern raised by consortium members was how faculty members can be supported to grow from their roles as conventional teachers to becoming learning facilitators. In the beginning of the case it was unclear what the role of a learning facilitator should be. Although some teachers enjoyed taking this role, some did not, and continued to give students the right answers instead of teaching them the tools needed to understand the whole system and from this derive their own solutions. Case leaders also discussed whether the facilitating role should be designated to specific individuals or whether it is the responsibility of all teachers in the course. To account for these individual preferences among teachers some cases reported they had two facilitators for each student group.

Engaging external stakeholders. The main areas of discussion at the second consortium meeting were how to meaningfully involve external food system stakeholders in knowledge creation and how the project could have positive impacts on the food system. One main concern raised was about engaging different stakeholder groups in the educational case studies, given that actors have different aims and expectations and often people speak different ‘languages’. The aim must be that everybody involved in transforming education should learn and derive something new from participating. To some consortium members it is still not always clear what role external stakeholders play and how they contribute.

*Group level*

Resistance among teaching peers. According to several members of the consortium, teachers are not always interested in alternative learning methods or are not willing to go outside their comfort zones. As an example, in one of the cases instructors are travelling from the outside to give lectures for one or two weeks in the course. For them to take the role as a learning facilitator was not appreciated by many teachers since this would mean to follow the students in their group projects during eight months, which is totally different from ‘parachuting in and making a short guest performance’ in the course.

*Institutional level*

Support from upper management. “We have the impression that it would be easier at NF partner universities, but it still would require a lot of work, and we would need a better way of communicating between teachers and heads of departments and the admin staff”. This quote was captured at the on-line meeting in 2020 and highlights one difficulty for the educational innovation to become institutionalized, i.e. become a part of the mainstream educational regime. One way case leaders tried to institutionalize their cases was to try to make the certificate course recognized as a credit course, which was not always accepted by the teaching institution.

Lack of rewards and credits. The institutional setting sometimes caused challenges to the implementation of the new learning approach. For example, in one case the teachers were only ready to spend a few hours in the field compared to the ten days of field activities that were needed to accomplish the action learning activities. Teachers and students not being rewarded for spending this amount of time in the field as well as the shortage of suitable teaching facilities at farmer training centers outside the university was particularly a challenge for one of the cases. 

Taking action learning on-line

The COVID-19 pandemic that hit in 2020 raised major challenges to teachers using action-oriented learning approaches, but also offered opportunities to develop creative methods for on-line learning. At the consortium conferences in 2020 and 2021, case leaders reported positive experiences of having the courses on-line, but at the same time they worried that lack of interaction with peers and stakeholders in real life might decrease learners’ experiential abilities. In some partner countries the infrastructure for was not enough developed in the countryside to successfully run courses on-line.

## Analysis and discussion

In this article we explore the development of a transformative innovation– the NEXTFOOD educational approach-- and its capacity to achieve outcomes that might contribute to a transition to sustainability action learning approaches in food and agricultural education. It is transformative because it is rooted in local teaching initiatives that are connected to a supracommunity for sharing ideas and experiences across different contexts ( Loorbach *et al.,* 2020). Transformative innovations can potentially contribute to transitions on a broader scale than regular innovation projects since they build on the collective efforts and experiences of many local initiatives (ibid.). The action research model pursued in this project favored a translocal development of the educational approach by supporting learning in local cases and at the same time stimulating a diffusion of experiences between teaching institutions in different geographical and cultural contexts. From a transition management perspective, a concern is how this development will continue after the project has ended. The individual cases will most likely continue on their chosen path on their own, but without having a connection to the learning network the transition will lose momentum and miss the opportunity for a broader impact on the wider educational sector. In this section we provide recommendations on how a transformative innovation can be firmly established in the regime of food and agricultural education.

The development of the NEXTFOOD educational approach represents the start of a productive transitioning process that will be ongoing. It began as a conceptual model for action learning education that was tested and further developed in several action research projects with promising contributions to the transition of the larger educational system. The outcomes of the cases resulted in changes in mindset among teachers and students, diffusion of educational practices and new structures at the teaching institutions. People working in local cases encountered several barriers an individual level (new roles for students, teachers and stakeholders), on a group level (resistance among teaching peers), and at the institutional level (no managerial support and inflexible administration). This is what one would expect from any transition experiment, that in similarity with NF, shows potential to contribute to a transition but with a high risk of failure due to many hindering forces ( Rotmans & Loorbach, 2009).

The promising contribution to educational transition was conveyed by the three mechanisms of deepening, broadening and scaling-up ( Van den Bosch, 2010). When it comes to deepening, the facilitated network learning process resulted in shared meanings and methods and improved the capacity to change the educational practices in courses and programs at the teaching institutions. In the cases, there was an intentional shift from the one-way transfer of knowledge through lectures to participatory action learning in the field. However, it was obvious that it takes a shift in mindset among the members of the network to go from a ‘content focus’ to a ‘process focus’, where attention is directed towards co-learning processes instead of only looking at their individual project outcomes and formal outputs such as reports. Implications for facilitators of translocal networks are to design a process with the right balance between content and process and promote regular reflective sessions for teachers, students and other actors involved in education where improvements in teaching methods are discussed. Activities for self-reflection are not common at teaching institutions today, and universities with ambitions to take an active role in societal change processes need to take steps towards becoming learning organisations ( Albrecht *et al.,* 2007). Higher education organisations are bound to tradition, and change therefore comes slowly and in small steps. There exists a fundamental paradox at the heart of higher education organizations: ‘they are institutions designed to teach, but not to teach themselves. Change, therefore, comes slowly and incrementally’ ( Stephens & Graham, 2010, pp 617).

When it comes to broadening, the educational experiment was repeated in several different locations and broadened in terms of types of learners, geographical and cultural contexts. It was also linked to different functions, i.e. pursuing education within different topics (agriculture, forestry and aquaculture) and for learners at different levels (high school, university and lifelong learning).

Some partners disseminated the NF educational model beyond the consortium by teaching faculty and staff at other institutes by means of the methods developed, which indicates that broadening was taking place. Some attempts were made to connect the cases directly to regional vocational schools and to international networks for sustainable education in agriculture but no firm connections were established during the project period. Broadening was encouraged by the formation of a supra-community connecting several multistakeholder educational cases and local learning processes. Being a part of such a community gave the case teams the necessary support to adopt the educational approach at their home institutions. The network learning process was important to reach shared goals and to align the various case activities. The cases that entered the project had from the beginning different levels of maturity and the broadening process implied a transfer of knowledge from the more experienced cases to the ones that recently adopted the approach.

The network learning model presented in this paper facilitated co-learning and collaboration by connecting innovative university initiatives into a community of practice and, in the long term, could support a transition of the educational system within agriculture, foods, and forestry. It is a useful instrument that can support the development of transformative innovations by allowing transition facilitators to study how learning outcomes from niche-experiments complement each other and contribute to enhancing a global sustainability trajectory. This local-global dynamics is based on a flow of knowledge and experience across individual transitions experiments, and one emergent property may be general lessons drawn from the collective experience ( Geels & Deuten, 2006). Literature on strategic niche management has emphasized the importance of learning from local experiments with novel ideas ( Hoogma *et al.,* 2017; Kemp *et al.,* 1998). Socio-ecological research that generates biological and social knowledge on the adoption of sustainable production methods in agriculture is a promising contribution to the transition literature ( Gaba & Bretagnolle, 2020; Teschner *et al.,* 2017). By facilitating reflexive learning and promoting productive interactions between researchers and stakeholders, it contributes to identify strategies for a sustainable transition. By linking several such experiments in a translocal learning process, e.g. by connecting to or developing an international network for annual conferences, publications and on-line communities, research outcomes could be combined and progress could be reached faster.

When it comes to scaling-up, the educational approach was more or less embedded in the institutional environment at the partnering institutions but still has to prove its merits in being accepted as a real alternative or complement to mainstream conventional education. Four years that the transition experiments lasted was not enough time to establish a firm foundation within mainstream education, which is not surprising since regime shifts are long-term changes. We identified a vast number of barriers that need to be addressed on the road to a transition to action-oriented sustainability education. Identified barriers were related to factors at teaching institutions, at group-level, and mindset of teachers, students and stakeholders. To support up-scaling, we can learn from transition management that strategic, tactical, operational and reflexive activities should be combined as these interact and reinforce each other ( Loorbach & Rotmans, 2010). Previous studies on change at universities suggest that there usually is more focus on operational and tactical levels, i.e. planning and running experiments, while less attention is given to the strategic and reflexive activities ( Thompson & Green, 2005).

It is necessary that sustainable education becomes a core mission of higher education institutions and that academic leaders reconsider the educational strategy, embracing a more integrated and holistic approach and co-curricular activities on campus and in the community ( Cortese & Hattan, 2010). Whereas bottom-up leadership and change initiatives driven by students and faculty are crucial to achieve the necessary change, top management leadership support is important to gain broad support and accomplish larger, more revolutionary educational transformation ( Lee & Schaltegger, 2014). In this study, one case received support from upper management to establish a center for agroecological research and education at the institute, which is one example of how managerial support can make the niche more stable. However, there was no evidence that the NF educational approach had been adopted at other programs at the partnering organisations or at neighboring teaching institutions. However, research outcomes and best practices disseminated by the end of the project might inspire others to drive change in education in the spirit of the NF project. Two main outcomes of the project were a roadmap and practical manuals for anyone who would like to initiate change in education. This may be useful for fresh initiatives that have to overcome the vast number of barriers identified in this project. 

We recognize that achieving an educational transition will require long-term commitment and involvement by participants in the project, as well as by stakeholders in the field. Hopefully the empirical knowledge on barriers and mechanisms of transition processes presented in this study may support the planning and implementation of future initiatives in the area of sustainability education.

## Conclusions

The NEXTFOOD case study showed how facilitation of an innovative educational approach in a network of local actors can be a key instrument to contribute to a transition in sustainability education. Results of a multi-dimensional learning process were observed as changes in how network members interpreted the educational approach, the implementation of new educational practices, and the structural changes taking place at the partnering institutions. These results were achieved also in cases where people in the institutional environment were far from supportive and willing to change. Changes in education were conveyed by the mechanisms deepening, broadening and scaling-up, and complemented rich descriptions of local cases with an analysis of the transition process across teaching institutions. This makes the results of our empirical research more generalizable and therefore outcomes could contribute significantly to successful implementation of future initiatives in sustainability education. Knowledge and experience flowed between the local projects and the educational approach was successfully repeated in several locations within different educational contexts, which could contribute to a regime change.

To what extent this education contributed to a food system transformation is difficult to assess, but the cases had a direct impact by the co-creation of knowledge in relation to real sustainability problems and by sharing nature-friendly practices in each community of practice. Indirectly the cases have an impact through the competences learners developed necessary to take responsible action for sustainability in their professional lives. To overcome the many barriers related to individuals, teams and institutions, bottom-up initiatives driven by teachers and students need support from educational management. Follow-up action research should investigate how the involvement of educational management in long-term goal formulation and envisioning may enhance scaling-up promising initiatives and contribute to a regime change.

## Data availability

### Underlying data

Zenodo: Educational transformation and network learning dataset – qualitative data from an international collaborative EU-project. DOI: https://doi.org/10.5281/zenodo.5810106. ( Melin *et al.,* 2021).

This project contains the following underlying data:

- SLU_V1.0_Consortium Workshop Notes_2021.12.29.docx. The file contains the compiled notes from the workshops organized at the four annual consortium conferences; Malmö, Sweden 2018; Budweis, Czech Republic 2019; On-line 2020; On-line 2021. (description of data in file).

Data are available under the terms of the Creative Commons Attribution 4.0 International license (CC-BY 4.0).
