# Supplementary material for: Facilitating transformative innovations in sustainability education
Source: Open Res Eur. 2022 Jun 15;2:22. Originally published 2022 Feb 18. [Version 2] doi: 10.12688/openreseurope.14407.2 (PMC10445915; doi:10.12688/openreseurope.14407.2)
Supplement: Peer review history

# Peer review: Reviewer response for version 2

**DOI:** 10.21956/openreseurope.16085.r29528

**Published:** 2022-06-29

**Authors:** David Gallar

**Affiliations:** 1 Institute of Sociology and Peasant Studies., University of Córdoba, Córdoba, Spain

The new version addresses in a more direct and concrete way the difficulties pointed out by the different actors of the project. This, in my opinion, is the greatest strength and usefulness of this text. Regarding the presentation of the project, the methodological framework and the results, this version, although less detailed, offers some much clearer answers. One could still try to clarify the successful strategies better, since they are not very clear, but the description of the limitations makes up for it.

If applicable, is the statistical analysis and its interpretation appropriate?

Partly

Is the study design appropriate and is the work technically sound?

Yes

Is the work clearly and accurately presented and does it engage with the current literature?

Partly

Are the conclusions drawn adequately supported by the results?

Partly

Are sufficient details of methods and analysis provided to allow replication by others?

Partly

Are all the source data and materials underlying the results available?

Partly

Reviewer Expertise:

Agroecological Education for Food Sovereignty - Peasant Studies - Rural Sociology

I confirm that I have read this submission and believe that I have an appropriate level of expertise to confirm that it is of an acceptable scientific standard.

# Peer review: Reviewer response for version 2

**DOI:** 10.21956/openreseurope.16085.r29527

**Published:** 2022-06-16

**Authors:** Helen Kopnina

**Affiliations:** 1 Northumbria University, Newcastle, UK; 2 The Hague University, Den Haag, The Netherlands

Good to see improvements in the revised version of this article.

If applicable, is the statistical analysis and its interpretation appropriate?

Yes

Is the study design appropriate and is the work technically sound?

Partly

Is the work clearly and accurately presented and does it engage with the current literature?

Partly

Are the conclusions drawn adequately supported by the results?

Partly

Are sufficient details of methods and analysis provided to allow replication by others?

Partly

Are all the source data and materials underlying the results available?

Yes

Reviewer Expertise:

environmental sustainability, environmental education, biodiversity conservation, sustainable business, degrowth

I confirm that I have read this submission and believe that I have an appropriate level of expertise to confirm that it is of an acceptable scientific standard.

# Peer review: Reviewer response for version 1

**DOI:** 10.21956/openreseurope.15550.r28835

**Published:** 2022-04-05

**Authors:** David Gallar

**Affiliations:** 1 Institute of Sociology and Peasant Studies., University of Córdoba, Córdoba, Spain

The text has a great strength which is the concept of “organizational learning”. However, it is not reflected how this project is linked to practices that have to do with sustainability or how the discussions and learning that had been shared in the NEXTFOOD´s meetings have been transferred to educational practices and agroecological content of each of the local actors. In the text, we have no record of the application of the pedagogical principles based on the student-centered education approach, nor what role other stakeholders have played, or the carrying out of field practices with farmers or communities. Nor is the interdisciplinarity of the project and the contents that apply shown.

 Regarding internal validity, the text presents in detail the methods of work and analysis of the research. However, despite the methodological apparatus and information collection techniques, it can be verified that the amount of fieldwork material analyzed and its analytical support is quite scarce since the code with the most quotes only has 39 quotes. In other words, it is surprising that having so much material gathered during the project after so many meetings, workshops, interviews, work in small and large groups, etc., the codes only achieve an average of 19 quotes (ranging between 7 and 39). In any case, beyond the small number of quotes, a finer qualitative analysis is not made.

 On the other hand, the presentation of the analysis fails to transfer the complexity of the actors involved: it only makes a brief and superficial comment on some elements, but which are not related to the methodological presentation around the 18 codes. In other words, a very brief presentation of topics is made (Teacher's role, Collaboration, Communication, Organization, External stakeholders) that leave out key elements of the dialogue and co-learning process (and it does not show how this is related to the 18 codes used).

 Network learning outcomes are addressed in another part of the text, although we are also unclear how these agreements between actors have been defined, or if there were nuances or discrepancies between types of actors or regions. Although this part does include some examples that allow us to better understand the complexity and internal discussions. It is missing to know if, and how, local actors contribute specific Bottom-up and Student-centered work practices and methodologies, and how they contribute to the coordination based on the co-production of knowledge and creation of more horizontal work dynamics 1) between themselves and 2) towards their own local actors in their territories.

 Regarding the content, the text is interesting, it provides an interesting theoretical and analytical approach on "organizational learning", but it would be important 1) to know the effective results in the learning and transformations of organizations, and 2) how that has been translated effectively in more horizontal and more agroecological practices and content (since right now the text could be talking about organizational learning carried out by any group of actors and with the content of any kind without any link to sustainability or agroecology). These deficiencies can surely be resolved with a better and deeper presentation of the enormous raw material generated in the work carried out throughout the project.

 The authors might consider the article in the references section of this review, of which I am a co-author, which can provide some insights into what are the key elements when learning processes are addressed, emphasizing who and how they participate, with what content, with what methodologies and with what horizon of learning transformation in the territory. ^1^

 p3: typo: tweleve

If applicable, is the statistical analysis and its interpretation appropriate?

Partly

Is the study design appropriate and is the work technically sound?

Yes

Is the work clearly and accurately presented and does it engage with the current literature?

Partly

Are the conclusions drawn adequately supported by the results?

Partly

Are sufficient details of methods and analysis provided to allow replication by others?

Partly

Are all the source data and materials underlying the results available?

Partly

Reviewer Expertise:

Agroecological education for Food Sovereignty

I confirm that I have read this submission and believe that I have an appropriate level of expertise to confirm that it is of an acceptable scientific standard, however I have significant reservations, as outlined above.

## References

[1] : Agroecological education for food sovereignty: Insights from formal and non-formal spheres in Brazil and Spain. *Journal of Rural Studies* .2021;88: 10.1016/j.jrurstud.2021.10.003 138-148 10.1016/j.jrurstud.2021.10.003

# Response

**Published:** 2022-06-07

**Authors:** Martin MELIN

Dear David, Thank you for your valuable comments on the manuscript. In the current revised version, we address the concerns of both reviewers related to how data was presented and discussed in relation to current literature. Most importantly, the manuscript has developed to better reflect the results of the network learning process, in particular how the learning was translated into new educational and sustainable practices and diffused among the participating teaching institutions. Examples are given on the application of the educational approach at the partnering institutions. To accomplish this, we introduced a new model for analysing our data that reflects the shift of three elements: mindset (culture), educational practices and structure.  The main focus of the article is how a new approach to sustainability learning can be developed and scaled-up in order to contribute to a fundamental change in the way education is done at universities today. The paper draws on the sustainability transition literature and includes papers on mechanisms for system transition. Outcomes could contribute to the successful implementation of future initiatives in sustainability education, but also make an input to the wider discussion on agrifood system transition. The study builds on twelve educational cases of different geographical and cultural contexts, which adds reliability and make the results relevant to other change initiatives in agrifood education as well as in other educational sectors. We hope you enjoy reading the new version of the article! Best wishes, The authors

# Peer review: Reviewer response for version 1

**DOI:** 10.21956/openreseurope.15550.r28836

**Published:** 2022-03-29

**Authors:** Helen Kopnina

**Affiliations:** 1 Northumbria University, Newcastle, UK; 2 The Hague University, Den Haag, The Netherlands

This article describes and analyses the NEXTFOOD Network, which is used as an example of how various goals are set, and how a transformation through their implementation is playing out. The methodology includes the action research approach, informed by several workshops organised at annual consortium conferences during the first three years of the project.

 Since the methodology is largely based on the action research approach, informed by several workshops organised at consortium conferences, it has limited replication/reliability value and tends to be case-based without expanding into contextual but theoretically important areas, e.g. root causes of environmental degradation related to food production. Also, as the methodology is related to very particular consortium conferences during the first three years of the project it does not reflect the ‘future potential’ or less insulated practices, e.g. the types of conferences that focus on issues related to climate change and/or biodiversity loss.

 It is not clear exactly how this project can concretely contribute to addressing the root causes of environmental crises, such as population and consumption/production growth, or how it concretely contributes to solving environmental challenges such as climate change and, in particular, biodiversity/extinction crises. While social and economic issues seem to take a centre stage in this project, very little is said about how the destruction of wild habitats by increasing agricultural output, e.g. animal farming, etc. is going to be reversed and how alternatives can be provided. There’s robust literature discussing how food production (technology) affects biodiversity loss and climate change, among other environmental and ethical issues (e.g. animal welfare in industrial animal farming or CAFOs) and there is also literature on these subjects in (environmental) education which is not reviewed/presented/built on in this article. Considering the missing literature the conclusions only seem to scratch the surface.

If applicable, is the statistical analysis and its interpretation appropriate?

Yes

Is the study design appropriate and is the work technically sound?

Partly

Is the work clearly and accurately presented and does it engage with the current literature?

Partly

Are the conclusions drawn adequately supported by the results?

Partly

Are sufficient details of methods and analysis provided to allow replication by others?

Partly

Are all the source data and materials underlying the results available?

Yes

Reviewer Expertise:

environmental sustainability, environmental education, biodiversity conservation, sustainable business, degrowth

I confirm that I have read this submission and believe that I have an appropriate level of expertise to confirm that it is of an acceptable scientific standard, however I have significant reservations, as outlined above.

# Response

**Published:** 2022-06-07

**Authors:** Martin MELIN

Dear Helen, Thank you for your valuable comments on the manuscript. In the current revised version, we address the concerns of both reviewers related to how data was presented and discussed in relation to current literature. Most importantly, the manuscript has developed to better reflect the results of the network learning process, in particular how the learning was translated into new educational and sustainable practices and diffused among the participating teaching institutions. Examples are given on the application of the educational approach at the partnering institutions. To accomplish this, we introduced a new model for analysing our data that reflects the shift of three elements: mindset (culture), educational practices and structure.  The main focus of the article is how a new approach to sustainability learning can be developed and scaled-up in order to contribute to a fundamental change in the way education is done at universities today. The paper draws on the sustainability transition literature and includes papers on mechanisms for system transition. Outcomes could contribute to the successful implementation of future initiatives in sustainability education, but also make an input to the wider discussion on agrifood system transition. The study builds on twelve educational cases of different geographical and cultural contexts, which adds reliability and make the results relevant to other change initiatives in agrifood education as well as in other educational sectors. We hope you enjoy reading the new version of the article! Best wishes, The team of authors
